# Radioactive Iodine Ablation Decrease Recurrences in Papillary Thyroid Microcarcinoma with Lateral Lymph Node Metastasis in Chinese Patients

**DOI:** 10.1007/s00268-017-4134-0

**Published:** 2017-07-24

**Authors:** Shuai Xue, Peisong Wang, Jia Liu, Guang Chen

**Affiliations:** Department of Thyroid Surgery, The 1st Hospital of Jilin University, Changchun, 130021 People’s Republic of China

## Abstract

**Introduction:**

We aimed to carry out a retrospective study from a single institution to determine whether radioactive iodine (RAI) ablation decreases the risk of recurrence of papillary thyroid microcarcinoma (PTMC) patients that presented with lateral lymph node metastasis (LLNM).

**Methods:**

We retrospectively analyzed a total of 6361 consecutive PTMC patients who initially underwent surgery for the treatment of thyroid carcinoma at the First Hospital of Jilin University, between January 2005 and February 2015. Altogether, 137 patients with PTMC with LLNM have been enrolled in our study.

**Results:**

The disease-free survival period was significantly shorter for the RAI (−) patients than for the RAI (+) patients (*p* = 0.0074 by the log-rank test). The disease-free survival rates at 5 and 10 years were 93.02 and 87.5%, respectively, in the RAI (−) group and 100 and 96.81%, respectively, in the RAI (+) group. CLNM ratio and LLNM ratio were factors identified for multivariate analysis by Cox’s proportional hazards method yielding risk ratios of 7.281 [CI 1.804–17.554; *p* = 0.010] and 1.157 [CI 1.0125–9.381; *p* = 0.048] in the RAI (−) group.

**Conclusion:**

Taken together, RAI may be beneficial for PTMC with LLNM, especially when CLNM ratio or LLNM ratio was greater than 0.5.

## Introduction

Papillary thyroid carcinoma (PTC) is the most common type of endocrine malignant cancers. Owing to a rapid increase in diagnosis of papillary thyroid microcarcinoma (PTMC), the incidence of PTC has been also increasing sharply [1]. This increase in PTMC resulted mainly from the increased detection of small nonpalpable nodules by high-frequent ultrasound and fine needle aspiration [2]. It has been reported that 38.5% among PTC in the USA were PTMC, while 48.8% in France and 35.7% in Shanghai, China [3, 4].

On the basis of the American Thyroid Association (ATA) guidelines in 2015, radioactive iodine (RAI) ablation was recommended for high-risk patients with gross extrathyroidal extension (ETE), distant metastasis, or incomplete tumor resection. For unifocal and multifocal PTMC without other adverse features, RAI was not routinely recommended. Because of low-quality evidence and the lack of long-term prognosis analysis, the guidelines just made week recommendation for intermediate-risk differentiated thyroid carcinoma [5].

Regarding to PTMC, several researchers found that PTMC, which had the characteristics of a malignant cancer, was often multifocal and prone to central lymph node metastasis (CLNM) [6–8]. Therefore, they suggested aggressive operation and even RAI ablation postoperatively for PTMC patients. In cases of differentiated thyroid carcinoma with LLNM, RAI was recommended by ATA because of the higher risk of persistent or recurrent disease, especially with the presence of extra-nodal extension or clinically positive or increasing number of macroscopic lymph nodes [5]. However, increasing studies demonstrated that the vast majority of PTMC were inert carcinomas, so aggressive surgery and RAI ablation were considered as overtreatments because they could not improve the recurrence and outcome [9, 10]. Moreover, such systematic review and meta-analysis of the effectiveness of RAI therapy for PTMC have been performed and conclude that for PTMC patients already treated by total thyroidectomy (TT), RAI ablation may not be helpful to decrease the incidence of thyroid cancer-related mortality or the 10-year recurrence of PTMC. Even for the intermediate-risk PTMC patients, RAI did not prevent recurrence [11]. Based on these disputes, the efficacy of RAI therapy for PTMC with lateral lymph node metastases (LLNM) is still unknown.

Thus, we aimed to carry out a retrospective study from a single institution to discuss whether RAI ablation decreases the risk of recurrence of PTMC patients that presented with LLNM.

## Materials and methods

### Patients

This study was approved by the Ethical Committee of the First Hospital of the Jilin University, and written informed consents were given to participants for their clinical records to be used in this study. We retrospectively analyzed a total of 6361 consecutive PTMC patients who initially underwent surgery for the treatment of thyroid carcinoma at the First Hospital of Jilin University, between January 2005 and February 2015. One hundred and thirty-seven patients recruited in the study met the following criteria: (a) patients’ information found in a hospital database and (b) patients with a postoperative pathological diagnosis of PTMC with LLNM. (c) Patients underwent total thyroidectomy with bilateral central lymph node dissection (CLND) and unilateral or bilateral lateral lymph node dissection (LLND). Patients were excluded from the study if pathological types of thyroid malignancies other than PTMC, age <18 years, high-risk patients with gross extrathyroidal extension (ETE), distant metastasis or incomplete tumor resection, lack of a preoperative fine needle aspiration (FNA) examination, had a history of neck radiotherapy, and with history of previous thyroid surgery, follow-up duration <6 months (loss to follow-up within 6 months, reoperation within 6 months after initial surgery, or residual malignancy or lymph node detected within 6 months after initial surgery). Finally, this study enrolled 137 patients with PTMC with LLNM.

### Initial treatment

Clinical diagnosis was initially made by examination of ultrasound (US) and FNA. US cervical mapping was done by an experienced, specially trained radiologist. For PTMC with LLNM, we performed TT and bilateral CLND with unilateral or bilateral LLND. This study included cases with both prophylactic and therapeutic CLND. CLND was performed to remove all lymph nodes and fibro-fatty tissue extending laterally from the medial border of the common carotid artery to the midline of the trachea and vertically from the hyoid bone to the thoracic inlet. All the patients underwent therapeutic LLND due to preoperative confirmation of LLNM which was diagnosed by US, CT, and/or FNA. In our institution, modified LLND including levels II–V with the preservation of the spinal accessory nerve, internal jugular vein, and sternocleidomastoid muscle is the standard treatment for LLNM. Level I dissection was not performed routinely unless indicated.

### Clinicopathological variable

Demographic data on patient clinical characteristics such as gender and age at diagnosis, as well as histopathological features (diameter of the largest tumor, total diameter of all tumors, multifocality, bilaterality, variants of PTMC, v-Raf murine sarcoma viral oncogene homolog B (BRAF) mutation, microscopic ETE, with chronic thyroiditis, regional lymph node metastasis, total and metastatic lymph node yield, TSH suppression and administered RAI), were recorded.

### Follow-up

Until 2011, TSH-suppressive hormonal therapy was applied to postoperative patients. We followed up all cases with physical examinations, serum thyroglobulin (Tg), Tg antibodies, US, and iodine-131 scans at 6-month intervals. There are no definite benefits that PTMC patients with LLNM could obtain from RAI ablation. As a result, surgeons must explain both the risks and benefits of RAI ablation and give informed consent forms to patients. The final decision was made based on physician or patient preference. When recurrence was suspected, patients underwent FNA with or without measurement of washout Tg levels and thyroid CT. In our study, recurrence was defined as the presence of tumor or metastatic lymph node at least 6 months after the initial surgery.

### Statistical analysis

To identify differences between groups for specific variables, SPSS version 22 software (SPSS Inc, Chicago, IL) was used for statistical analysis, which was performed by Pearson’s Chi-square test or Student’s *t*-test. Survival curves were drawn by Kaplan–Meier method and statistically analyzed by the log-rank test. To characterize PTMC with LLNM, univariate analysis and multivariate analysis were performed by Cox’s proportional hazards method for disease-free survival and the risk factors. A *p* value <0.05 was considered statistically significant.

## Results

Altogether, 137 patients of PTMC with LLNM have been enrolled in our study, in which 94 cases underwent with RAI while other 43 cases did not. The clinicopathological characteristics of the enrolled patients (*n* = 137) are shown in Table 1. Administered RAI was found to be different significantly between two groups (*p* = 0.000). There were no significant differences between the two groups in terms of age, gender, microscopic ETE, diameter of the largest tumor, total diameter of all tumors, multifocality, bilaterality, presence of chronic thyroiditis, variants of PTMC, BRAF mutation, LLND, total and metastatic lymph node yield, CLNM ratio, LLNM ratio, and TSH suppression.

**Table 1:** Clinicopathological characteristics of PTMC patients with LLNM according to RAI ablation

	RAI (−) (*N* = 43)	RAI (+) (*N* = 94)	*p* value
Gender			
Female	30	71	
Male	13	23	0.477
Age			
<45 yr	9	21	
≥45 yr	34	73	0.853
Diameter of largest tumor			
≤0.5 cm	14	19	
>0.5 cm	29	75	0.250
Total diameter of all tumors			
≤0.5 cm	2	6	
0.5–1.0 cm	14	18	
1.0–2.0 cm	11	34	
>2.0 cm	16	36	0.329
Multifocality			
Absent	12	22	
Present	31	72	0.571
Bilaterality			
Absent	18	35	
Present	25	59	0.606
Microscopic ETE			
Absent	3	8	
Present	40	86	1.000
With chronic thyroiditis			
Absent	35	77	
Present	8	17	0.942
Variants of PTMC			
Classical	39	84	
Follicular	2	3	
Diffuse sclerosing	0	3	
Tall cell	1	0	
Solid	1	1	
Insular	0	0	
Oxyphilic	0	1	
Columnar	0	1	
Others	0	1	0.608
BRAF mutation			
Absent	6	16	
Present	37	78	0.650
LLND			
Unilateral	40	86	
Bilateral	3	8	1.000
Number of total CLN	5.17 ± 1.02	6.01 ± 1.14	0.998
Number of metastatic CLN	1.43 ± 0.09	2.82 ± 0.77	0.223
Number of total LLN	11.2 ± 2.11	10.98 ± 2.07	1.000
Number of metastatic LLN	4.93 ± 1.11	4.37 ± 1.04	0.573
CLNM ratio			
<0.5	33	79	
≥0.5	10	15	0.305
LLNM ratio			
<0.5	34	81	
≥0.5	9	13	0.294
TSH supperssion			
Yes	37	85	
No	6	9	0.446
Average follow-up period (years)	6.75 ± 2.11	6.07 ± 1.97	0.816
Administered RAI			
0 mCi	43	0	
≤30 mCi	0	2	
30–100 mCi	0	15	
100–150 mCi	0	59	
>150 mCi	0	18	0.000

*ETE* extrathyroidal extension, *LLND* lateral lymph node dissection, *PTMC* papillary thyroid microcarcinoma, *CLNM ratio* central lymph node metastasis ratio (positive lymph node number/sum lymph node number), *LLNM ratio* lateral lymph node metastasis ratio (positive lymph node number/sum lymph node number)

**Fig. 1:**
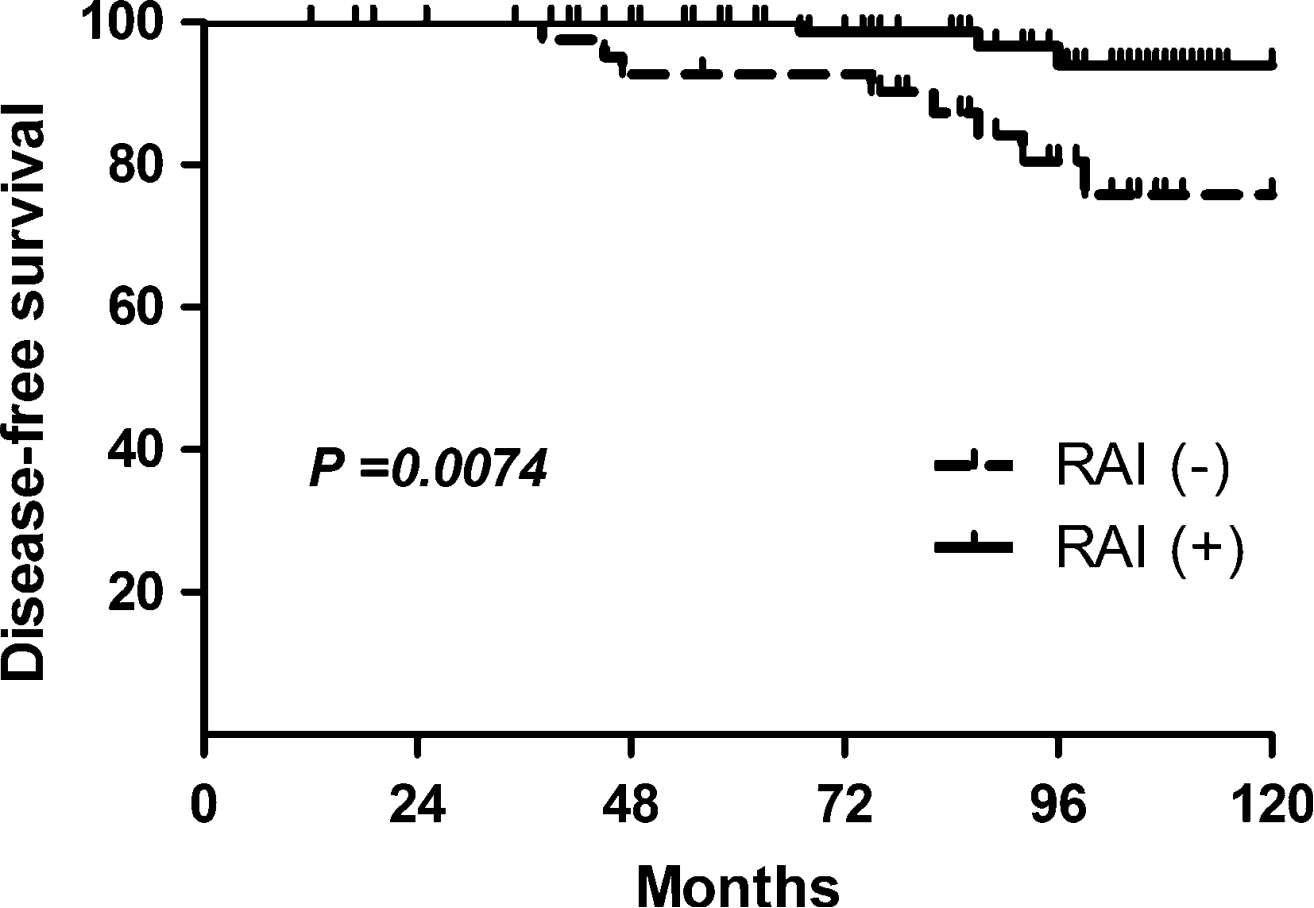
Disease-free survival period was significantly shorter for the RAI (−) patients than for the RAI (+) patients (*p* = 0.0074 by the log-rank test)

The disease-free survival period was significantly shorter for the RAI (−) patients than for the RAI (+) patients (*p* = 0.0074 by the log-rank test) (Fig. 1). Details of recurrent cases are shown in Table 2. The disease-free survival rates at 5 and 10 years were 93.02 and 87.5%, respectively, in the RAI (−) group and 100 and 96.81%, respectively, in the RAI (+) group. Because nobody died of thyroid cancer, disease-specific mortality did not differ statistically between groups (data not shown).

**Table 2:** Characteristics of recurrent cases

Patient	Age/ sex	Max tumor size(mm)	M E T E	Multifocality	Bilaterality	BRAF	Sum diameter of all tumors	CLNM ratio	LLNM ratio	RAI	Tg (ng/ml)	Initial operation	Time to recurrence (month)	Site of locoregional recurrence	Size of recurrent lymph node or tumor (cm)
1	44/F	0.8	+	+	+	+	1.3	+	+	−	4.24	TT + CLND + uLLND	38	Contralateral central	0.9
2	35/F	0.5	−	+	−	+	0.8	+	+	−	7.12	TT + CLND + uLLND	47	Thyroid bed	0.8
3	77/F	0.4	+	+	+	+	0.9	+	+	−	18.43	TT + CLND + uLLND	102	Ipsilateral lateral	1.1
4	39/M	0.8	−	−	−	+	0.8	−	+	−	23.94	TT + CLND + uLLND	82	Ipsilateral lateral	2.1
5	37/F	0.6	+	+	−	+	1.4	+	+	−	47.11^a^	TT + CLND + uLLND	45	Ipsilateral lateral	2.3
6	69/F	0.4	+	+	+	+	1.2	+	+	−	10.32	TT + CLND + bLLND	111	Thyroid bed	1.2
7	61/F	0.4	−	+	−	+	0.7	+	−	−	2.19	TT + CLND + uLLND	75	Contralateral central	1.4
8	45/M	0.7	+	+	+	+	1.5	+	−	−	15.11	TT + CLND + uLLND	92	Ipsilateral lateral	2.4
9	58/M	0.9	+	+	−	+	1.6	+	+	−	2.98^a^	TT + CLND + uLLND	103	Thyroid bed	0.8
10	56/F	0.8	+	+	−	+	1.2	+	−	+	62.03	TT + CLND + uLLND	99	Contralateral lateral	2.1
11	68/F	0.7	+	+	+	+	1.3	−	+	+	29.72	TT + CLND + uLLND	110	Ipsilateral lateral	1.3

*METE* microscopic extrathyroid extension, *CLNM* central lymph node metastasis (CLNM ratio ≥0.5 was defined as positive while <0.5 as negative), *LLNM* lateral lymph node metastasis (LLNM ratio ≥0.5 was defined as positive while <0.5 as negative), *RAI* radioactive iodine ablation, *TT* total thyroidectomy, *CLND* central lymph node dissection, *uLLND* unilateral lymph node dissection, *bLLND* bilateral lymph node dissection, *Tg* serum thyroglobulin at diagnosis of recurrence

^a^ Tg without TSH suppression

**Fig. 2:**
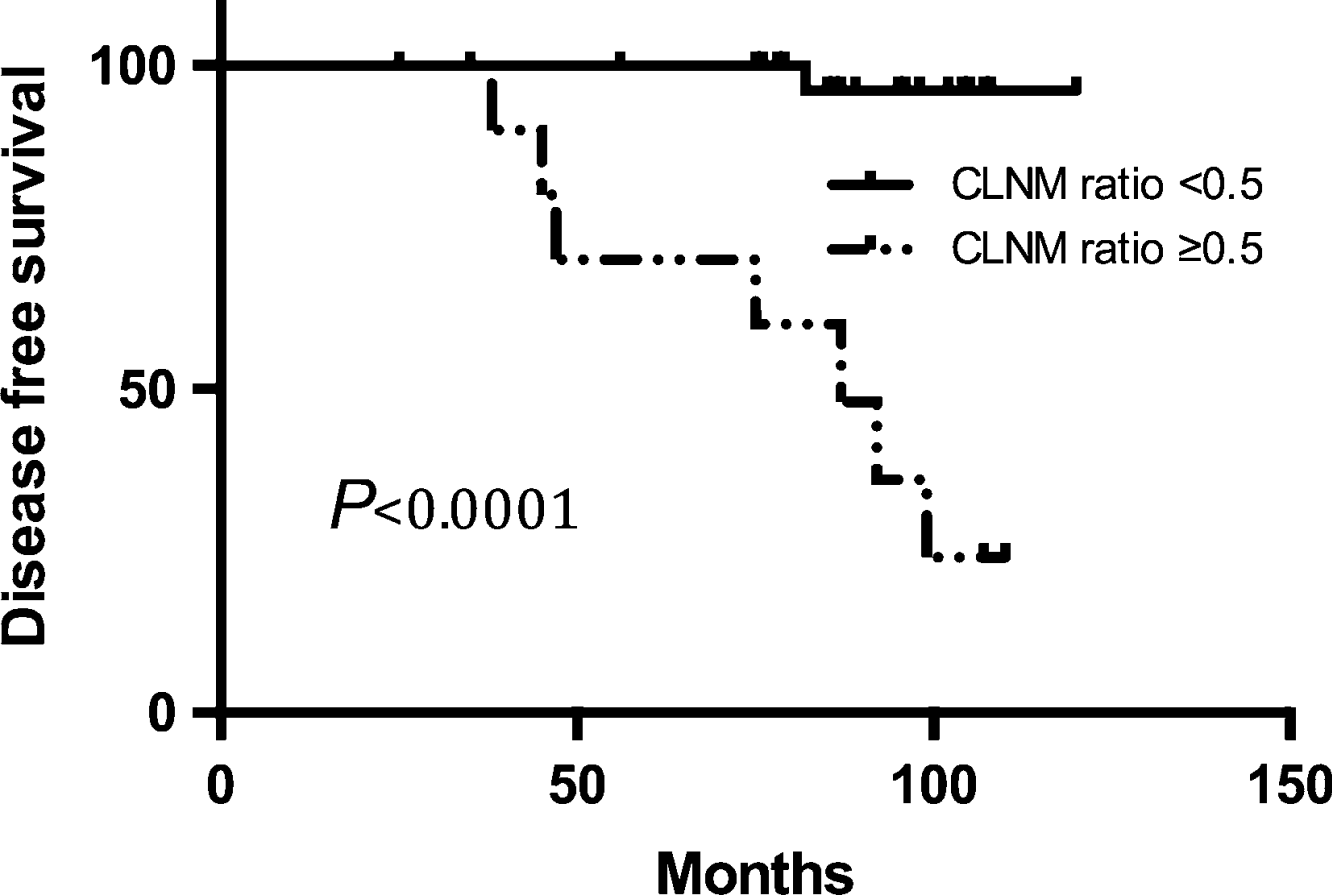
Disease-free survival period was significantly shorter for the CLNM ratio ≥0.5 patients than for the CLNM ratio <0.5 patients (*p* < 0.0001 by the log-rank test)

**Fig. 3:**
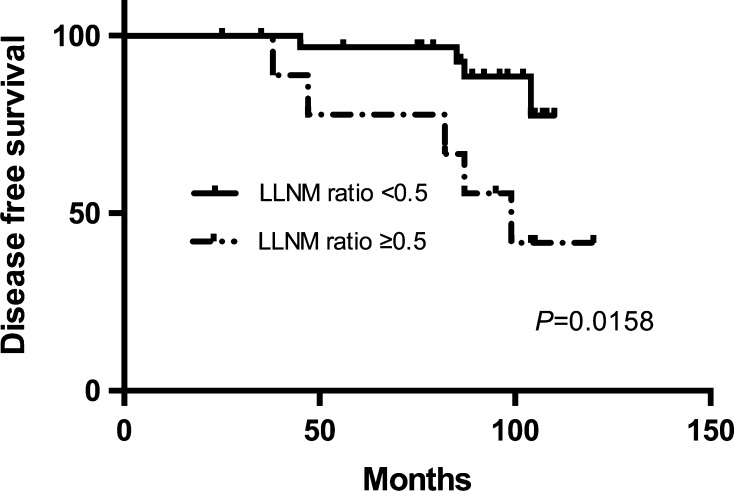
Disease-free survival period was significantly shorter for the LLNM ratio ≥0.5 patients than for the LLNM ratio <0.5 patients (*p* = 0.0158 by the log-rank test)

Univariate analysis by Cox’s proportional hazards method showed CLNM ratio and LLNM ratio to be risk factors for recurrence of RAI (−) patients (Table 3). Age, gender, microscopic ETE, diameter of the largest tumor, total diameter of all tumors, multifocality, bilaterality, presence of chronic thyroiditis, variants of PTMC, BRAF mutation, and LLND were not predictors of recurrence. The risk ratio for CLNM ratio and LLNM ratio was 12.948 [confidence interval (CI) 2.610−64.229; *p* = 0.002] and 8.321 [CI 1.234–32.321; *p* = 0.031] in the RAI (−) group. No other risk ratios obtained were of interest. CLNM ratio and LLNM ratio were factors identified for multivariate analysis by Cox’s proportional hazards method (Table 4) yielding risk ratios of 7.281 [CI 1.804–17.554; *p* = 0.010] and 1.157 [CI 1.0125–9.381; *p* = 0.048] in the RAI (−) group. The disease-free survival period was significantly shorter for the CLNM ratio ≥0.5 and LLNM ratio ≥0.5 patients (*p* *<* 0.0001 and *p* = 0.0158 by the log-rank test) (Figs. 2, 3). Accordingly, CLNM ratio and LLNM ratio were shown to be independent predictors of disease-free survival.

**Table 3:** Subgroup univariate analysis by Cox’s proportional hazards method for disease-free survival

Factors analyzed	RAI (−) patients	
	Risk ratio (CI)	*p* value
Age (years)	0.034 (0.000–48.227)	0.362
Male gender	1.170 (0.279–4.911)	0.830
Microscopic ETE	0.794 (0.096–6.551)	0.830
Diameter of largest tumor	0.621 (0.148–2.609)	0.515
Sum diameter of all tumors	1.624 (0.388–6.802)	0.507
LLND bilaterality	2.070 (0.254–16.892)	0.497
Multifocality	1.051 (0.211–5.239)	0.952
Bilaterality	0.443 (0.106–1.855)	0.265
With chronic thyroiditis	0.895 (0.109–7.360)	0.918
BRAF mutation	1.443 (0.177–11.780)	0.732
Variants of PTMC	0.595 (0.073–4.842)	0.628
CLNM Ratio	12.948 (2.610–64.229)	0.002
LLNM Ratio	8.321 (1.234–32.321)	0.031

*ETE* extrathyroidal extension, *LLND* lateral lymph node dissection, *PTMC* papillary thyroid microcarcinoma, *CLNM ratio* central lymph node metastasis ratio (metastatic central lymph node/harvested central lymph node), *LLNM ratio* lateral lymph node metastasis ratio (metastatic lateral lymph node/harvested lateral lymph node)

**Table 4:** Subgroup multivariate analysis by Cox’s proportional hazards method for disease-free survival

Factors analyzed	RAI (−) patients	
	Risk ratio (CI)	*p* value
CLNM ratio	7.281 (1.804–17.554)	0.010
LLNM ratio	1.157 (1.0125–9.381)	0.048

*CLNM ratio* central lymph node metastasis ratio (metastatic central lymph node/harvested central lymph node), *LLNM ratio* lateral lymph node metastasis ratio (metastatic lateral lymph node/harvested lateral lymph node)

## Discussion

There is a wide awareness that radioactive treatment can eliminate residual normal or tumor thyroid cells which further leads to decrease recurrence [12]. Although generally safe, RAI has several potential side effects, classified as early and late complications [13, 14]. More severely, when compared to the general population, female PTC patients who treated with RAI had increasing risk of second primary malignancy, and this risk was not apparent significantly in those without RAI [15, 16]. The medical decision process for referral to treatment with RAI should therefore incorporate a careful analysis of the benefits and risks for this therapy, particularly in the case of younger patients. Moreover, increasing reports published recently have demonstrated that RAI could not decrease disease-free survival and disease-specific mortality in PTMC patients, even in the intermediate-risk DTC [10, 11]. Thus, we performed this retrospective study to evaluate the function of RAI in PTMC with LLNM (belong to intermediated-risk DTC) and explore the prognostic factors for recurrence.

In our study, the disease-free survival period was significantly shorter for the RAI (−) patients than for the RAI (+) patients (*p* = 0.0074 by the log-rank test). Creach et al. [17] reviewed the therapy and outcome of 407 patients with PTMC, and they found that the 5-year disease-free survival for patients treated with RAI was 95.0 versus 78.6% (*p* < 0.0001) for patients not treated with RAI. In their study, patients with lymph node metastasis who did not receive RAI had a 5-year disease-free survival of 42.9 versus 93.2% (*p* < 0.0001) for patients who received RAI, which was consistent with our research. Moreover, AL-Qahtani et al. [18] concluded in their study that adjuvant RAI therapy improves disease-free survival in PTMC patients with aggressive histopathologic variants, multifocality, ETE, lymphovascular space invasion, tumor size (>0.5 cm), and lymph node involvement. However, in recent years, several studies suggested that RAI did not prevent recurrences in patients with PTMC. In Hye Jeong Kim’s study, we have huge concerns about the selection bias since the RAI (−) group was only 30 cases compared with the RAI (+) group of 450 patients in the subgroup of intermediated risk [10]. Furthermore, 74% cases in RAI (−) group were TNM stage I while only 47% in RAI (+) group. In another systemic review and meta-analysis, they found that there was almost no positive treatment effect of RAI ablation noted for patients with PTMC. But the dose of RAI was unavailable in most studies and the follow-up period was different among researches. Consequently, these conclusions were not powerful enough to demonstrate efficacy of RAI in PTMC.

Massive prognostic parameters for recurrence of PTMC have been identified in previous studies, such as male gender, age <45, ETE, multifocality, primary tumor size, BRAF, T3 tumor, and lymph node metastasis [19–27]. In our subgroup study analysis, CLNM ratio and LLNM ratio were factors identified for multivariate analysis by Cox’s proportional hazards method (Table 4) yielding risk ratios of 7.281 [CI 1.804–17.554; *p* = 0.010] and 1.157 [CI 1.0125–9.381; *p* = 0.048] in the RAI (−) group. The disease-free survival period was significantly shorter for the CLNM ratio ≥0.5 and LLNM ratio ≥0.5 patients (*p* *<* 0.0001 and *p* = 0.0158 by the log-rank test) (Figs. 2, 3). Accordingly, CLNM ratio and LLNM ratio were shown to be independent predictors of disease-free survival. Like a previous study, when the CLNM ratio is higher than 0.44, there is an increased risk of locoregional recurrence mostly in the lateral neck [21]. Additionally, Kim et al. [25] also found LLNM was a risk factor for recurrent PTMC. Accordingly, we believe that RAI may be beneficial for PTMC with LLNM, especially when CLNM ratio or LLNM ratio was greater than 0.5.

Our study has some limitations. The first limitation is the small number of patients in the study population; Due to the incidence of PTMC with LLNM is low, and we want long duration of follow-up, we just summarized patients between January 2005 and February 2015. The second limitation is that until 2011, TSH-suppressive hormonal therapy was applied to postoperative patients. At that time, there was no guideline which we could follow to recommend TSH-suppressive hormonal in China. Despite these limitations, our study has important implications for PTMC management and provides significant information for PTMC guideline formation.

## Conclusion

Taken together, RAI may be beneficial for PTMC with LLNM, especially when CLNM ratio or LLNM ratio was greater than 0.5. A multi-centric pragmatic randomized controlled clinical trial with large population is needed to assess the effects on recurrence, quality of life, and cost-effectiveness outcomes of different treatments and follow-up regimen for PTMC with LLNM.
